# Utilizing Peripheral Nerve Blocks for Pain Management in Pediatric Patients during Embolization and Sclerotherapy for Vascular Malformations

**DOI:** 10.3390/children11030368

**Published:** 2024-03-20

**Authors:** Matthew Kocher, Maria Evankovich, Danielle R. Lavage, Sabri Yilmaz, Senthilkumar Sadhasivam, Mihaela Visoiu

**Affiliations:** 1 Department of Anesthesiology and Perioperative Medicine, UPMC Children’s Hospital of Pittsburgh, University of Pittsburgh Medical Center, 4401 Penn Avenue, Pittsburgh, PA 15224, USA; kochermr@upmc.edu (M.K.); sadhasivams@upmc.edu (S.S.); 2 Department of Anesthesiology and Perioperative Medicine, University of Pittsburgh Medical Center, Pittsburgh, PA 15213, USA; evankovichmr2@upmc.edu; 3 Department of Anesthesiology and Perioperative Medicine, University of Pittsburgh School of Medicine, Pittsburgh, PA 15213, USA; lavagedr@upmc.edu; 4 Department of Radiology, UPMC Children’s Hospital of Pittsburgh, Pittsburgh, PA 15224, USA; sabri.yilmaz@chp.edu

**Keywords:** peripheral nerve block, vascular malformation, embolization therapy, sclerotherapy, opioid consumption, perioperative pain

## Abstract

Vascular anomalies are a diverse group of abnormal blood vessel developments that can occur at birth or shortly afterward. Embolization and sclerotherapy have been utilized as a treatment option for these malformations but may cause moderate-to-severe pain. This study aims to evaluate the utilization of peripheral nerve blocks in opioid consumption, pain scores, and length of stay. A retrospective chart review was conducted at the UPMC Children’s Hospital of Pittsburgh for all patients who underwent embolization and sclerotherapy between 2011 and 2020. Patient data were collected to compare opioid consumption, pain scores, and length of stay. In total, 854 procedures were performed on 347 patients. The morphine milligram equivalent per kilogram mean difference between groups was 0.9 (0.86, 0.95) with a *p*-value of <0.001. The pain score mean ratio was −1.17 (−2.2, −0.1) with a *p*-value of 0.027. The length of stay had an incident rate ratio of 0.94 (0.4, 2) and a *p*-value of 0.875. By decreasing opioid consumption and postoperative pain scores, peripheral nerve blocks may have utility in patients undergoing embolization and sclerotherapy while not clinically increasing the length of stay for patients. Their use should be individualized and carefully discussed with the interventional radiologist.

## 1. Introduction

Vascular anomalies are disorders of the endothelium that can affect each part of the vasculature (capillaries, arteries, veins, or lymphatics). Over the past two decades, many changes and updates have occurred in vascular anomalies, including their histopathology. A significant step was the adoption of the Mulliken and Glowacki proposal of dividing vascular anomalies into tumors and malformations by the International Society for the Study of Vascular Anomalies in 1996 [1]. Vascular tumors demonstrate endothelial proliferation and may rapidly enlarge postnatally. Malformations are errors in vascular development and have stable endothelial turnover; lesions are named based on the malformed primary vessel (capillary, arterial, venous, and lymphatic) [2]. 

Embolization and sclerotherapy are two types of treatment and have been used for several decades to treat vascular malformations. In the catheter embolization of arteriovenous malformations, medications or synthetic materials called embolic agents are placed through a catheter to eliminate abnormal connections between arteries and veins. Many types of embolic agents are used for occluding the vessels. Particulate agents, including polyvinyl alcohol (PVA) and gelatin-impregnated acrylic polymer spheres (Embospheres), are suspended in liquid and injected into the bloodstream to block small vessels. These agents are used to block blood vessels permanently. Various-sized metallic coils or mechanical devices made of stainless steel or platinum block large arteries and arteriovenous fistulas. Liquid sclerosing agents like alcohol and sodium tetradecyl sulfate destroy blood vessels and vascular malformations. Filling a blood vessel or vascular malformation with these liquid agents causes blood clots to form, closing the abnormal vascular channels. Liquid glue is another embolic agent that hardens quickly and forms a cast when injected into the target channel that needs to be closed off. 

Sclerotherapy, in which a solution is injected into a vascular malformation or lymphatic cyst percutaneously, causing it to collapse, scar, and fade, remains the primary treatment for venous malformations and lymphatic malformations. The sclerosant agents used are sotradecol 3% and absolute alcohol for venous malformations. Doxycycline and bleomycin treat macrocystic lymphatic malformations and microcystic lymphatic malformations, respectively.

A concern following both therapies is pain management. Significant pain following embolization is common, often necessitating narcotic medication use [3]. One way to counter the use of narcotics in patients undergoing embolization therapy and reduce pain is through peripheral nerve blocks [4]. Using peripheral nerve blocks in pediatric patients undergoing embolization and sclerotherapy for vascular malformations can provide several potential benefits. However, there is limited information on the effect of peripheral nerve blocks on postoperative pediatric pain in patients undergoing embolization therapy. 

This study investigates the utility and practicality of peripheral nerve blocks for pain control following embolization and sclerotherapy in pediatric patients with vascular malformations. We hypothesize that single-injection peripheral nerve blocks provide adequate perioperative analgesia for patients undergoing vascular malformation therapy by reducing morphine milligram equivalents (MMEs), postoperative pain scores, and length of stay.

## 2. Materials and Methods

### 2.1. Data Collection

This retrospective chart review study was conducted at UPMC Children’s Hospital (CHP) of the University of Pittsburgh Medical Center between September 2011 and October 2020. The University of Pittsburgh Institutional Board Review approved it, waiving the written consent requirement. Consent was obtained from patients who had images used for the manuscript. Electronic medical records were reviewed for all embolization and sclerotherapy procedures performed at the CHP during the study period. Patient demographics, vascular malformation procedures, perioperative opioid consumption, peripheral nerve block information and medication administered, pain scores, and the duration of the hospital stay were all collected from patient charts. Patients were excluded from the review if no embolization or sclerotherapy was performed during the procedure.

### 2.2. Embolization and Sclerotherapy Procedures

Embolization and sclerotherapy procedures were performed at the UPMC CHP following the induction of general anesthesia. Upon obtaining vascular access, the interventional radiologist used fluoroscopy to identify the correct vessels for treatment. Embolization and sclerosing agents were used as described above. Upon the completion of the therapy, patients emerged from anesthesia and recovered in the PACU.

### 2.3. Grouping of Patients for Analysis

Patients were categorized into two groups: those who received standard medical therapy including opioids and those who received standard medical therapy plus peripheral nerve blocks. The perioperative pain medications given to the patients were left to the discretion of the anesthesiologist and practitioners who were involved in the patient’s care.

### 2.4. Peripheral Nerve Block Procedure

The Acute Pain Service identified the patients who required a vascular malformation procedure and could be candidates for nerve blocks and contacted the interventional radiologist. The decision to perform a nerve block was based on the location and size of the vascular malformation, the probability of the patient experiencing moderate-to-severe pain, previous patient pain experience, and opioid consumption.

The block selection was based on the malformation location. All the blocks were performed following induction of general anesthesia prior to sclerotherapy or embolization therapy, under ultrasound guidance only (24 cases), a combination of ultrasound and nerve simulator (27 cases), and one ankle block without either ultrasound or nerve stimulator.

### 2.5. Outcome Parameters Analyzed

Embolization and sclerotherapy procedure types were classified based on anatomic locations as follows: (1) head and neck, (2) thorax and abdomen, (3) upper extremity, (4) lower extremity, and (5) multiple anatomical locations.

Pain scores were collected and recorded in the patient’s chart using validated scales, including NRS, Wong BAKER, and FLACC [5,6,7]. Opioid administration was carried out following the institutional protocol based on the PACU nursing assessment and pain scores. Opioid consumption was calculated based on MMEs and patient weight. Intraoperatively, fentanyl was used on induction, and acetaminophen was given at the end of the procedure. In the Pediatric Anesthesia Care Unit (PACU), fentanyl, morphine, and hydromorphone were the intravenous opioids given to patients with moderate-to-severe pain based on their pain scores. Intravenous methadone was given to four patients following procedures for patients who did not receive a nerve block. Few patients also received ketorolac or ibuprofen. The dosing of the medications was based on institutional guidelines.

### 2.6. Statistical Analysis

Descriptive statistics were calculated. Categorical variables were described using counts and percentages. Continuous variables were described using medians and inner quartile ranges. Histograms were constructed to visualize continuous distributions. Comparative testing was performed between those who received blocks and those who did not. Box plots were created to visualize differences between procedure location, block usage, outcomes of morphine milligram equivalents (MMEs), and postoperative pain scores. MMEs and the length of stay (LOS) were log-transformed to help visualize differences and normalize highly skewed distributions. Log transformation removed 9% of MME records that had 0 MME. 

Absolute standardized mean differences were used to compare differences between the groups [8]. This analysis method was chosen over traditional univariate methods (*t*-tests, chi-squared) as there could be multiple records per person, and several individuals had records for embolization procedures with and without blocks, making *t*-tests and chi-squared inappropriate. A value of >0.2 was considered a difference.

Due to repeated measures and crossover of block usage per patient, mixed models were chosen to test for differences between block groups. Separate models were fit for outcomes of MMEs, MMEs/weight, pain scores, and LOS. MMEs and MMEs/weight were highly skewed and were log-transformed to improve model fitting. All MME values were right-shifted by 1 to avoid loss of missing data due to log transform. Pain scores were nearly normal and modeled using linear mixed models. LOS was rounded to the nearest day, treated as count data, and modeled using negative binomial mixed models. Fixed effects were fit for receiving block treatment. Random intercepts were fit per patient to control for within-patient variation. Adjusted models were also fit using gender and age as adjustment covariates. Age was skewed and was z-transformed to assist in model fitting.

Missing data were removed from all denominators, continuous summaries, and statistical testing. Data management was performed in SAS version 9.4 (SAS Institute, Cary, NC, USA; 2016) and R software (version 4.2.1, R Core Team, Vienna, Austria, 2022). Descriptive statistics, testing, and visualization were performed in R. 

## 3. Results

In total, 854 procedures were performed on 347 patients during the specified period (Table 1). Of the 854 vascular malformation procedures, 802 did not utilize a peripheral nerve block for postoperative pain control. Most patients had between one and three therapies, while one patient had over twenty procedures performed. Of the 347 patients, the weighted percentage of patients who were female was 60.5%. The mean age for patients undergoing therapy was 11.7 years old (SD 8.13), with a mean weight of 45.65 kg (SD 26.5). The most common location for vascular malformation therapy was the head and neck region (*n* = 338, 39.6%), followed by lower extremity (*n* = 286, 33.5%) and upper extremity (*n* = 108, 12.6%). 

Most patients received sotradecol 3% (64.1%) or doxycycline (24.5%) for their therapy. Other less common agents used for therapies included dehydrated ethanol and bleomycin (Table 2). One patient received Onyx liquid embolic agent, one with micron Embospheres, another with an Amplazer vascular plug, and one with Hilal pushable coils. The median amount of each agent is described in Table 3, focusing on the extremities and trunk of the body.

**Figure 1:**
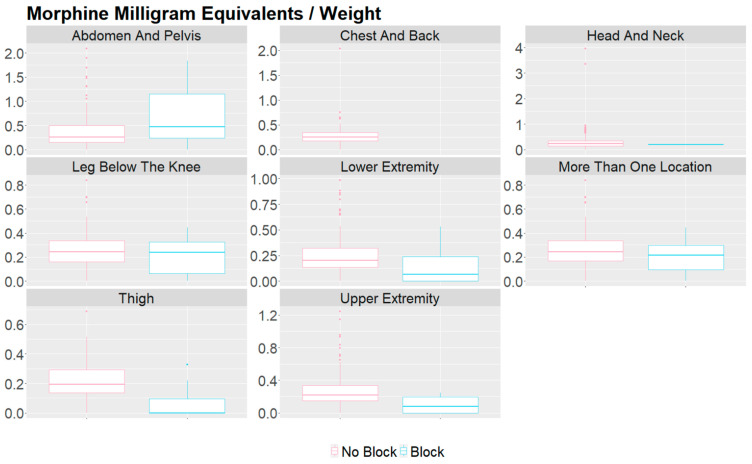
Box plot of MME/kg comparison between groups that received a peripheral nerve block and those that did not for vascular malformation procedures in various body regions. The *y*-axis is morphine milligram equivalents per kilogram. The points located beyond the whiskers are outliers and are located anywhere from (Minimum to Q1 − 1.5*IQR) and (Q3 + 1.5*IQR to Maximum).

**Figure 2:**
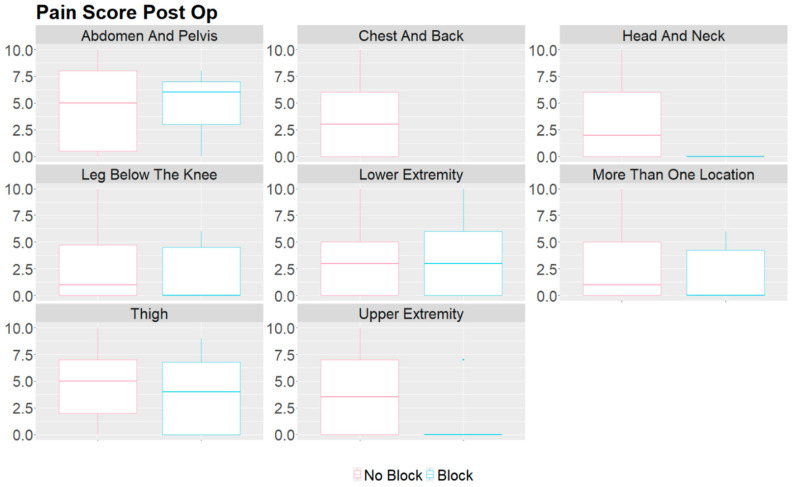
Box plot of MME/kg comparison between groups that received a peripheral nerve block and those that did not for vascular malformation procedures in various body regions. The *y*-axis is the postoperative pain score on a scale of 0–10.

Table 4 describes the various locations for therapy about the usage of opioids, postoperative pain, and length of stay. The abdomen and pelvis therapies had an opioid utilization of 0.26 mg/kg and a median pain score of 5. Lower extremity therapies had an opioid utilization of 0.19 mg/kg with a median pain score of 3 (Table 4). The usage of opioids and postoperative pain scores between the groups in different regions of the body are visualized in Figure 1 and Figure 2. Table 5 describes the utilization of nerve blocks for all patients who underwent vascular malformation therapy.

The MME/kg geometric mean ratio between the groups was 0.9 with a confidence interval of (0.86 and 0.95) and a *p*-value of <0.001 (Table 5). The pain score mean difference was −1.17 with a confidence interval of (−2.2, −0.1) and a *p*-value of 0.027 (Table 6). The follow-up of patients undergoing peripheral nerve blocks showed that patient/parent satisfaction was 9.7 on a 10-point scale. The length of stay had an incident rate ratio of 0.94 with a confidence interval of (0.4, 2) and a *p*-value of 0.875 (Table 6). One patient was removed from testing due to the LOS being an outlier of over 300 days. 

After data collection for this study, we performed an additional 83 peripheral nerve blocks on 48 patients. Unfortunately, these patients were not included in this data set. We did not encounter any complications from the nerve blocks performed.

## 4. Discussion

**Figure 3:**
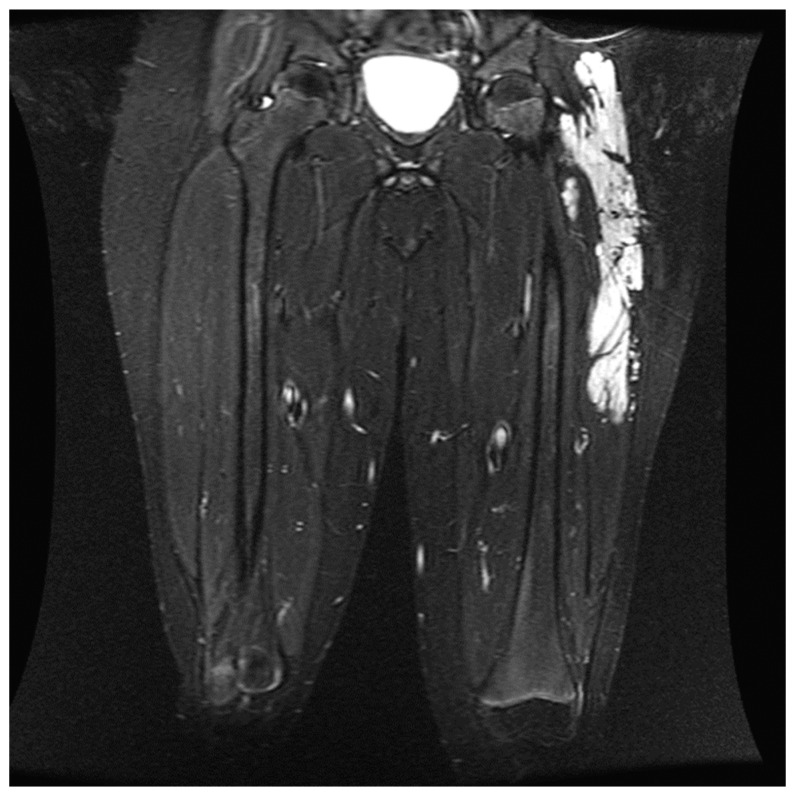
Coronal T2-weighted fat-suppressed image shows an extensive hyperintense slow flow vascular malformation consistent with a venous malformation involving the intramuscular and subcutaneous planes of the left lateral thigh and hip. During the specified range of dates, the child received four procedures. Each procedure was performed with a lumbar plexus nerve block for postoperative pain control.

**Figure 4:**
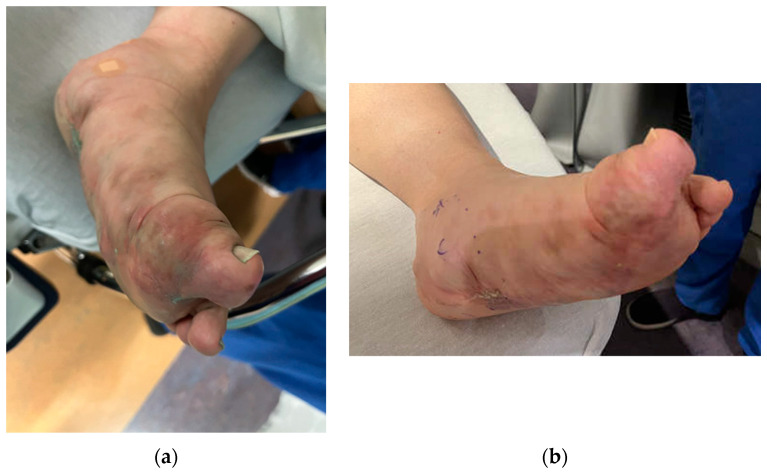
(**a**) Vascular malformation of the left foot showing the change in coloration and swelling of the limb before intervention. Peripheral nerve blocks were utilized to provide analgesia in the postoperative setting. (**b**) Upon repeat intervention, the patient’s foot showed improvement in swelling and discoloration. Patient consent was obtained for the use of the images.

**Figure 5:**
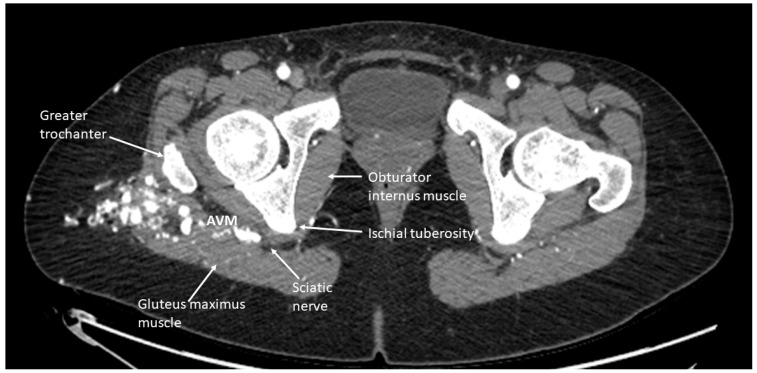
CT scan imaging of a right hip arteriovenous malformation with involvement close to the sciatic nerve. This patient had undergone several sclerotherapy procedures and received a quadratus lumborum nerve block for postoperative pain management. Following one therapy, the patient was noted to have a sciatic nerve injury.

The peripheral nerve blocks in pediatric patients undergoing embolization and sclerotherapy for vascular malformations can provide several potential benefits as indicated below:

1. They can effectively relieve pain during and after vascular malformation therapy and decrease the opioids required during general anesthesia and in the immediate postoperative period. By targeting specific nerves or nerve plexuses responsible for pain perception in the affected area, peripheral nerve blocks can reduce the need for systemic pain medications, which may have side effects and potential complications, such as respiratory depression, constipation, nausea, itching, and altered mental status following surgery [9,10]. This study showed an association between reduced MME/kg usage and pain scores in the block group, indicating that nerve blocks are associated with reduced MMEs in selected patients. This finding is consistent with using peripheral nerve blocks in other surgeries [11,12,13].
2. Minimizing pain and discomfort can improve the child’s and parents’ overall experience and reduce anxiety. Almost all patients and parents who completed the satisfaction questionnaires (26) were very satisfied with the postoperative pain control provided (8–10 on a 10-point scale). Only one patient rated their satisfaction with the pain control as a 7. Most of these patients returned for repeated sclerotherapy and received another block for postoperative pain control.
3. Reducing the use of opioids and general anesthesia can lead to faster recovery times and shorter hospital stays for pediatric patients. This can particularly benefit outpatient procedures, such as vascular malformation therapies. In our study, there was no difference in the length of stay between the two groups, indicating that the nerve blocks did not facilitate a faster discharge at home despite better pain control in the block group. It is essential to mention that there were no complications from the nerve blocks, and most patients could go home the same day as the procedure. However, some patients who experienced severe pain when a previous procedure was performed with no nerve block were very anxious to be discharged and stay overnight on the day of surgery. In addition, in many cases, enoxaparin therapy was initiated on the procedure’s day and required hospitalization.
4. When performing peripheral nerve blocks in pediatric patients undergoing embolization or vascular therapy, it is essential to consider the child’s location and size of the vascular malformation. Vascular malformations are frequently seen in children, with the most common area being in the head and neck [14]. Our retrospective study had similar results, with 39.6% of the vascular malformations treated in the study in the head and neck region. Most head and neck malformations are not amenable to a peripheral nerve block due to the location of the sensory nerves in the face and neck. The upper and lower extremities were the second and third most common sites for therapy, aligning with the most common areas of malformations. These locations are much more amenable to peripheral nerve blocks as nerve blocks have proven safe and effective in treating pain in the extremities. Careful consideration must be given when determining if a patient should receive a peripheral nerve block for therapy (Figure 3). In this study, the proceduralist requested a peripheral nerve block for patients based on the location of the malformation and the likelihood of the treatment causing severe pain. Some of the nerve blocks performed were for patients who had previously received therapy without nerve blocks but developed severe pain in the postoperative setting (Figure 4).
5. When administering peripheral nerve blocks in pediatric patients, it is essential to consider potential complications from the nerve block and injecting the sclerosing/embolizing agents. The risk of nerve injury from nerve blocks is low, around 2 to 4 per 10,000 blocks in children, but it should still be considered in all patients undergoing this type of therapy [15]. One patient developed a sciatic nerve injury following therapy with a peripheral nerve block. The malformation was located in the hip and close to the sciatic nerve (Figure 5). A quadratus lumborum nerve block was performed intraoperatively for postoperative pain control [16]. The patient made a full neurologic recovery after several months. The nerve injury was likely due to the therapy and unrelated to the peripheral nerve block. However, determining if the malformation is close to a nerve should raise concerns that signs of a nerve injury may be initially masked by a peripheral nerve block. As previously reported for other blocks by Pickle et al. [17] encountered no bleeding complications at the block sites after enoxaparin administration. Continuous patient monitoring during and after the procedure is vital to ensure their safety. Close observation for potential complications related to the nerve block, such as nerve injury, is necessary. At the UPMC CHP, we performed home phone call follow-ups for all the patients who received peripheral nerve block follow-up. Some of our patients required pre- and post-procedure anticoagulation therapy, and we investigated if any of our patients developed any hematoma at the needle injection site. In addition, some patients require a longer time to recover from peripheral nerve blockade.

This study does have some limitations. Notably, this was a single-center, retrospective chart review study. While there was some follow-up with the patients to determine how satisfied they were with their postoperative pain management, not all patients had a follow-up assessment for their pain management. Another limitation was the sample size for groups. This study had 854 therapies, but only 52 peripheral nerve blocks were performed. Blocks were performed based on proceduralist recommendation rather than randomization. Some data points were missing from patient charts, and not all patients had follow-up evaluations for pain score assessment at home after peripheral nerve blocks. While there was a difference in MMEs/kg between groups, a larger sample size would be needed to strengthen the results of this study. Still, it is important to mention that we did not encounter any complications, and the patients and their families reported increased satisfaction with their pain control. 

It is essential to offer and educate the patient (if age-appropriate) and their family about the nerve block procedure that can be performed for vascular malformation therapy and expected pain after the procedure. This can help alleviate anxiety and improve pain control and safety after the procedure. In some cases, alternatives to peripheral nerve blocks, such as intravenous and oral opioid medication and acetaminophen, should be offered to the patient to improve post-procedure pain control.

## 5. Conclusions

In summary, peripheral nerve blocks can be a valuable adjunct in managing pain and reducing the need for general anesthesia in pediatric patients undergoing embolization therapy for vascular malformations. However, their use should be individualized and carefully discussed with the interventional radiologist, considering the patient’s age, malformation size and location, and the need for anticoagulation therapy. The primary goal should always be to provide safe and effective pain management while minimizing risks and ensuring the child’s comfort and well-being. This study aims to provide insight and further investigation into the use of peripheral nerve blocks for perioperative pain management by other institutions with randomized control trials and strengthen these findings.

## Figures and Tables

**Table 1:** Patient characteristics along with general clinical findings. Absolute standardized mean differences indicate any variation between the two groups.

Variable	Total Mean (SD)	No Block Mean (SD) *n* = 802 (335 Distinct Patients)	Block Mean (SD) *n* = 52 (24 Different Patients)	Absolute Standardized Mean Differences
Age	11.7 (8.13)	11.6 (8.1)	14 (8.3)	0.3
Weight (Kg)	45.65 (26.5)	45.3 (26.8)	50.7 (20)	0.23
Height (cm)	140.71 (33.23)	139.9 (33.7)	153 (20.6)	0.47
BMI	20.54 (5.52)	20.5 (5.6)	20.7 (5)	0.04
Female—*n* (%)	517 (60.5%)	491 (61.2%)	26 (50%)	0.23

**Table 2:** Description of agents used for treatments. The percentage of each agent used in the therapies is shown with the total number (*n*).

Sclerosing Agent Used for Therapy	Percentage of Sclerosing Agent Used for Therapy (Number of Therapies)	Percentage of Sclerosing Agents Used for Treatment without Nerve Block (Number of Therapies)	Percentage of Sclerosing Agent Used for Therapy with Nerve Block (Number of Therapies)
Bleomycin	5.6 (47)	5.9 (47)	0 (0)
Bleomycin, doxycycline	0.7 (6)	0.8 (6)	0 (0)
Doxycycline	24.5 (207)	25.6 (203)	7.8 (4)
Ethanol	1.5 (13)	1.4 (11)	3.9 (2)
Onyx	0.4 (3)	0.4 (3)	0 (0)
Sotradecol	64.1 (542)	63 (500)	82.4 (42)
Sotradecol, bleomycin	0.1 (1)	0.1 (1)	0 (0)
Sotradecol, doxycycline	0.8 (7)	0.9 (7)	0 (0)
Sotradecol, ethanol	2.2 (19)	2 (16)	5.9 (3)

**Table 3:** Quantitative analysis for the dose of sclerosing/embolizing agent used in each location. Median and IQR were utilized due to the wide range of dosing. Locations are grouped due to sample size in some places.

Descriptive Table of Sclerosing Agent Doses by Surgery Location			
Location	Sclerosing Agent	Procedure in Location—*n*	Procedure in Location—Median (IQR)
All extremities	Bleomycin (units)	16	9 (5.75–11)
	Doxycycline (mg)	50	167.5 (62.5–300)
	Ethanol (mL)	3	5 (2.75–5)
	Sotradecol 3% (mL)	324	10 (7–12)
Lower extremity	Bleomycin (units)	1	2.5 (2.5–2.5)
	Doxycycline (mg)	19	150 (47.5–200)
	Ethanol (mL)	1	0.5 (0.5–0.5)
	Sotradecol 3% (mL)	137	10 (7–12)
Chest, back, abdomen, and pelvis	Bleomycin (units)	9	15 (8–15)
	Doxycycline (mg)	80	200 (100–500)
	Ethanol (mL)	4	12 (4.25–19)
	Sotradecol 3% (mL)	64	9.5 (5.88–12)

**Table 4:** Evaluation of each body region about opioid usage, postoperative pain, and length of stay. Aggregate values for both groups are included.

Procedure Location	Morphine Milligram Equivalents (MMEs) Median (IQR)	MMEs/Weight Median (IQR)	Post-Surgery Pain Score Median (IQR)	Length of Stay (Days) Median (IQR)
Abdomen and pelvis	9.25 (3.75–29.06)	0.26 (0.16–0.5)	5 (0.25–8)	0.34 (0.23–2.16)
Chest and back	6.38 (3–15)	0.25 (0.17–0.35)	3 (0–6)	0.25 (0.22–0.38)
Head and neck	7.5 (3.75–15)	0.24 (0.15–0.34)	2 (0–6)	0.25 (0.21–0.97)
Lower extremity	7.5 (3.75–15)	0.19 (0.11–0.31)	3 (0–5)	0.33 (0.25–1.18)
Upper extremity	7.5 (3.75–15)	0.21 (0.14–0.32)	3 (0–7)	0.27 (0.21–1.18)
More than one location	8.25 (4.5–15)	0.24 (0.17–0.34)	1 (0–5)	0.25 (0.21–1.13)

**Table 5:** Peripheral nerve block characteristics for all patients who underwent nerve blocks.

Block Performed	Age Mean	Weight (kg) Mean	MMEs/kg Mean	Ropivacaine (mg/kg) Mean	Pain Score Median	LOS Median
Cervical plexus	5	21.2	0.25	0.40	7	0.46
Digital	8	36.6	0	0.27	0	0.25
Erector spinae	6	18	0	2.78	0	0.23
Femoral	13.7	56.83	0.11	2.60	0	0.88
Femoral and lateral femoral cutaneous	17.25	52.68	0.28	2.55	3.5	0.29
Femoral and sciatic	9	33.6	0.21	2.08	0	0.94
Interscalene	13.5	73.45	0.11	1.36	0	0.73
Lateral femoral cutaneous	12.25	41.54	0.08	1.63	3	0.425
Lumbar plexus	20	64.56	0.30	1.12	7	1.13
Sciatic	13.1	51.28	0.16	1.90	0	2.17
Supraclavicular	10	34.33	0.12	1.45	0	0.53

**Table 6:** Comparison of patient populations who received a peripheral nerve block before vascular malformation therapy and those who did not. ^a^ Primary independent variables in models were categorical variables for receiving blocks (estimate shown) or not (reference level). Adjusted covariate estimates are omitted from the table for simplicity. ^b^ Models adjusted by gender and height z scores. ^c^ All MME outcomes were shifted by 1 unit to avoid data loss due to the log transformation of zero values. ^d^ One outlier of LOS > 300 was removed prior to modeling LOS.

Outcome	Covariate ^a^	Estimate (95% CI)	*p*-Value	Adjusted Estimate ^b^ (95% CI)	Adjusted *p*-Value
Log-Linear Mixed Models					
Morphine equivalents MMEs ^c^	Block–Received	0.43 (0.33, 0.58)	<0.001	0.43 (0.33, 0.56)	<0.001
Morphine equivalents MMEs/weight ^c^	Block–Received	0.9 (0.86, 0.95)	<0.001	0.91 (0.86, 0.96)	0.001
Linear Mixed Models					
Pain	Block–Received	−1.17 (−2.2, −0.1)	0.027	−1.25 (−2.3, −0.2)	0.018
Negative Binomial Mixed Models					
LOS ^d^ (rounded to nearest day)	Block–Received	0.94 (0.4, 2)	0.875	1.25 (0.6, 2.7)	0.572

## Data Availability

The data presented in this study are available on request from the corresponding author. The data are not publicly available due to University policy.
